# An Invasive Case of Left-Sided Endocarditis Caused by Pseudomonas aeruginosa in a Patient with History of Intravenous Drug Abuse

**DOI:** 10.7759/cureus.1717

**Published:** 2017-09-27

**Authors:** Ankit Mahajan, Mohammad Amer, Ahmad Awan, Fasil Tiruneh, Charu Gandotra, Bryan Curry

**Affiliations:** 1 Department of Internal Medicine, Howard University Hospital; 2 Cardiology, Howard University Hospital

**Keywords:** infective endocarditis, infective endocarditis, pseudomonas, intravenous drug abuse

## Abstract

Infective endocarditis, caused by Pseudomonas aeruginosa, is rarely seen in clinical practice. It has been reported mainly in intravenous drug abusers (IVDA). We present a case of a 63-year-old male who presented with abdominal pain and fever. Computed tomography (CT) abdomen showed splenic and renal infarct. The blood culture grew Pseudomonas aeruginosa. A transthoracic echocardiogram showed aortic insufficiency with 13 mm mobile vegetation. The patient was started on ceftazidime and tobramycin and, later on, surgery was done for aortic valve replacement. His stay was complicated by multiple hemorrhagic emboli in the brain. This case highlights the importance of the early diagnosis and management of infective endocarditis caused by Pseudomonas aeruginosa.

## Introduction

Infective endocarditis caused by Pseudomonas aeruginosa is rarely seen in clinical practice. It has been reported mainly in intravenous drug abusers (IVDA). Untreated, it carries a very high mortality rate of 80%, necessitating early diagnosis and intervention [1]. The clinical outcome of right-sided endocarditis has improved, with cure rates reaching 84%, while that of the left side remains at 33% [2]. A new pattern of infective endocarditis in IVDA is emerging, characterized by more frequent left heart involvement, more severe clinical course, and an early need for surgery. This suboptimal outcome is partly due to increasing resistance to beta-lactams and aminoglycosides. We report a challenging case of aortic valve endocarditis caused by Pseudomonas aeruginosa complicated with embolic stroke and atrial flutter and describe its medical management and outcome.

## Case presentation

**Figure 1:**
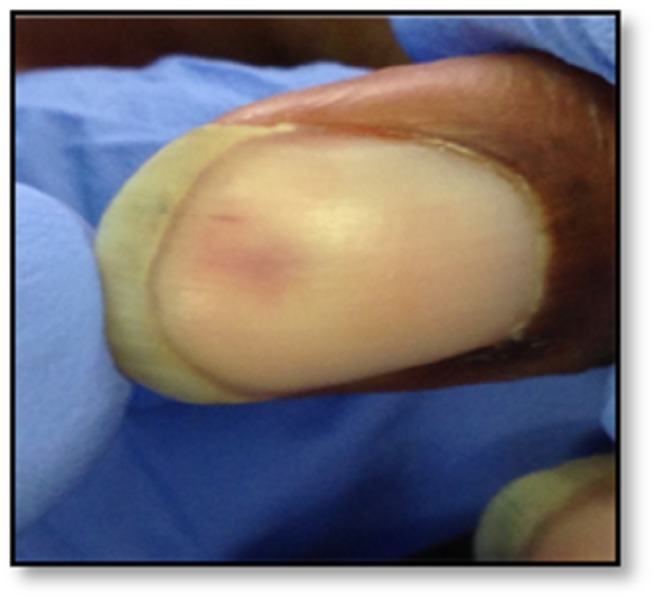
Splinter hemorrhage in our patient

A 63-year-old African American male with past medical history significant for hypertension, hyperlipidemia, and intravenous drug abuse presented with periumbilical abdominal pain of three days' duration. Abdominal pain was non-specific, gradual in onset, constant, without any aggravating or alleviating factors, and associated with nausea. The patient reported a history of intravenous drug abuse. The rest of the history was unremarkable. On physical examination, the patient was febrile (100.9^o^F) and tachycardic (103 beats per minute). An abdominal exam revealed tenderness in the left lower quadrant and voluntary guarding with no rigidity or rebound. Cardiovascular examination was significant for diastolic murmur over the right sternal border. Splinter hemorrhages were found in the nail bed of the patient (Figure 1).

**Figure 2:**
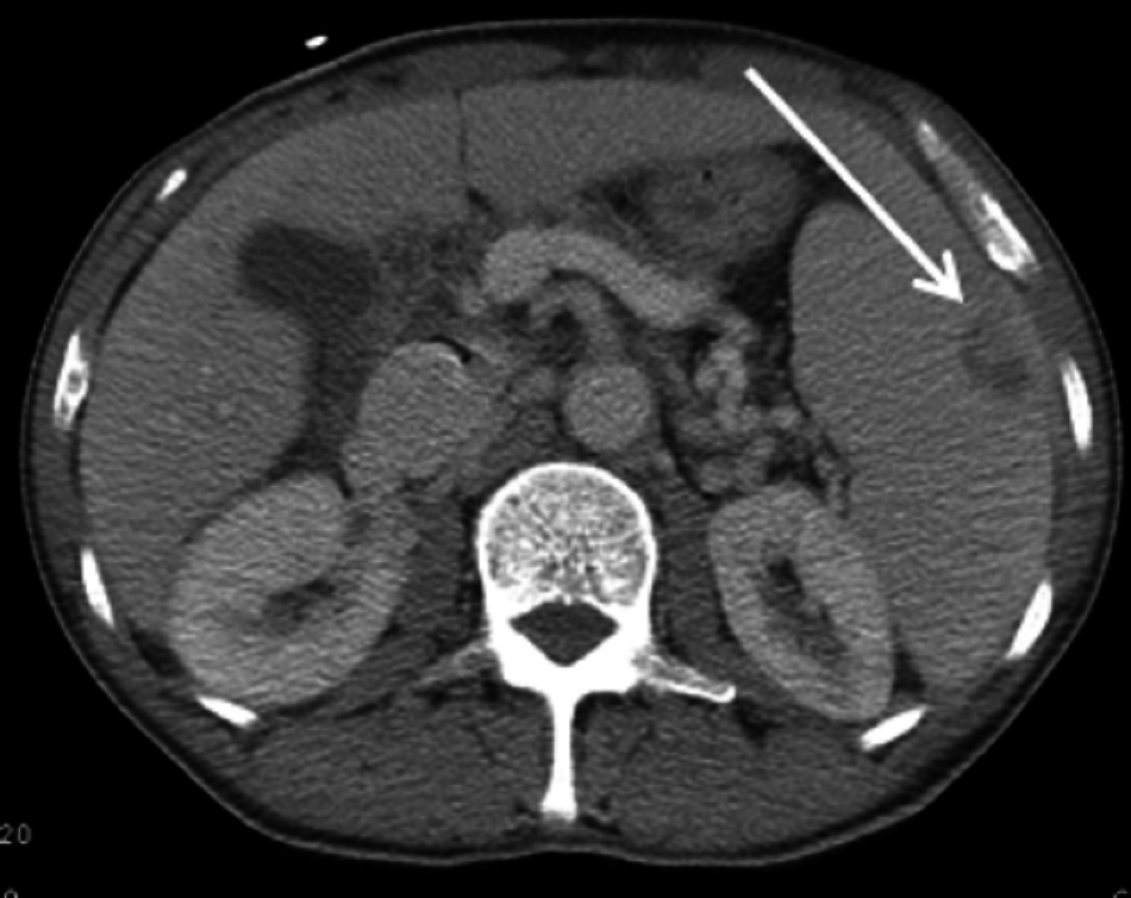
Showing wedge-shaped splenic infarct (arrow)

**Figure 3:**
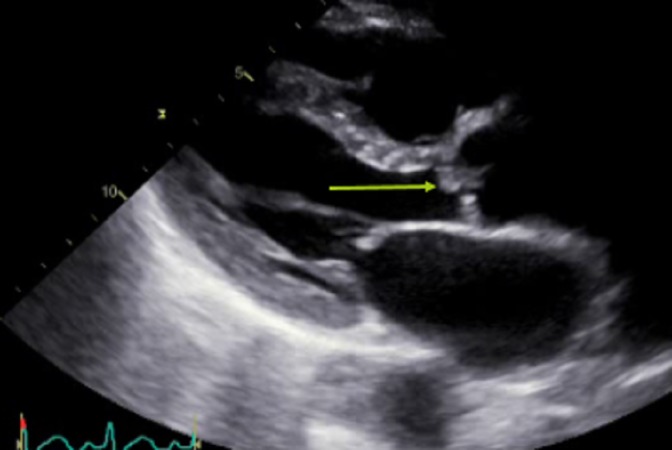
Echo showing an oscillating mass along the aortic valve (arrow)

Laboratory findings were significant for a leukocyte count of 14,000 cells per microliter with normal electrolytes and liver function tests. To further evaluate the etiology of abdominal pain, computed tomography (CT) abdomen was done, which showed a small, wedge-shaped infarct in spleen and both kidneys (Figure 2). The patient was initially assessed as sepsis with unclear etiology and started on broad-spectrum antibiotics (vancomycin and piperacillin-tazobactam). The blood culture grew gram-negative rods. As part of the sepsis workup, blood culture and urine culture were done. Because of the patient’s history of intravenous drug abuse with findings of splenic infarct, there was a concern for infective endocarditis. A transthoracic echocardiogram was done, which showed severe aortic insufficiency with an oscillating echo-density, which was measured to be 1.3 cm (Figure 3). The organism was later on identified as “Pseudomonas aeruginosa,” and the patient was switched to ceftazidime and tobramycin.

**Figure 4:**
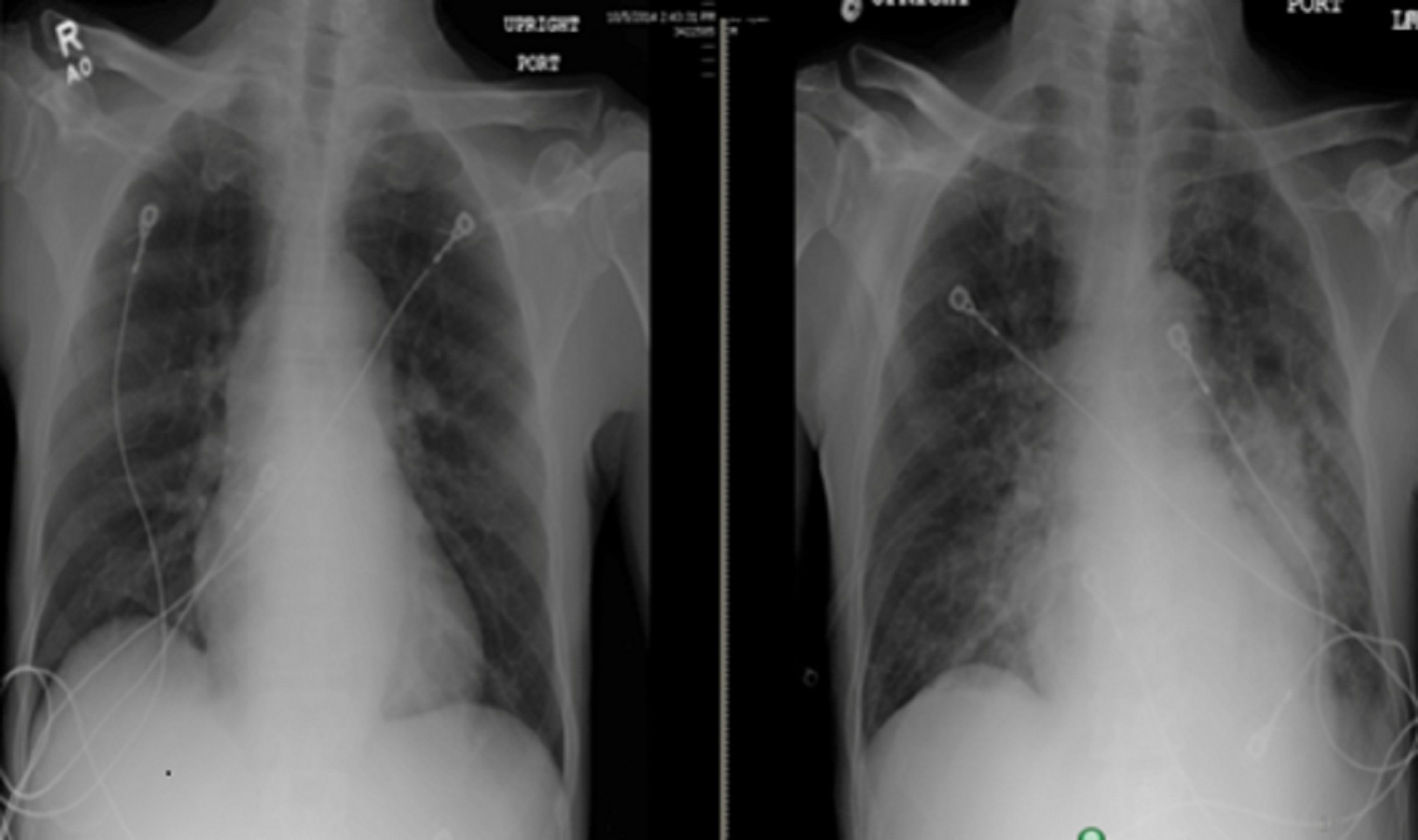
Chest X-ray before (left) and after (right) the surgery showing the development of pulmonary edema after the surgery

On day three of admission, the patient developed severe shortness of breath. On physical examination, he had crackles up to the middle of the lungs. A chest X-ray showed florid pulmonary edema (Figure 4). In view of decompensated heart failure in the setting of aortic insufficiency, cardiothoracic surgery was consulted for possible valve replacement. He underwent aortic valve replacement with a porcine mosaic valve.

**Figure 5:**
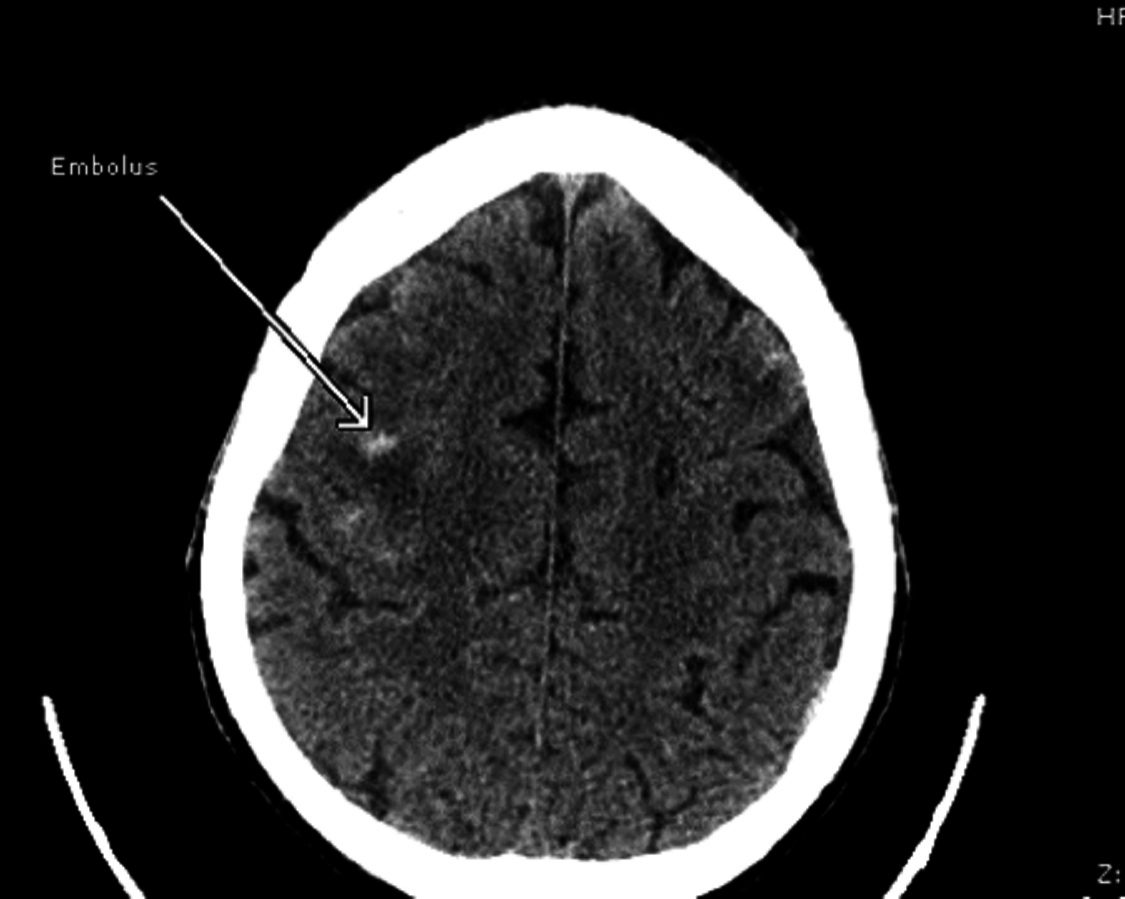
Computerized tomography of the head showing hemorrhagic embolus

On post-op day one, the patient developed left-sided weakness. A computed tomography (CT) scan of the head revealed multiple hemorrhagic embolic (Figure 5). The patient was managed according to the hemorrhagic stroke protocol. His condition improved, and he became euvolemic; however, left-sided weakness persisted. He was discharged to long-term acute care hospital (LTACH) for the completion of an antibiotics course for a total duration of six weeks.

## Discussion

The incidence of infective endocarditis (IE) has been rising steadily in the United States in the past decade. Between 2000 and 2011, incidence has increased from 11 per 100,000 to 15 per 100,000 [3]; however, the exact incidence cannot be determined because of changing criteria. IE, generally, as a whole and specifically in intravenous drug users (IVDU) most commonly involves the right side of the heart. Involvement of the left side of the heart is less common.

IE was initially recognized as a complication of intravenous drug use in the 1950s [4]. IE due to drug use is more common in males, and it occurs at a younger age when compared to non-users [5]. Staphylococcus aureus is the most common cause of IE among this group [6]. Streptococcus and Enterococcus are the next common organisms. Rarely, fungi and gram-negative rods can also cause endocarditis in IVDU. Pseudomonas aeruginosa is a rare cause of IE and mostly causes right-sided endocarditis. There are a few cases of left-sided endocarditis in patients with intravenous drug abuse but mostly these people had shunts like atrial septal defect [1]. The clinical outcome of right-sided endocarditis has improved with cure rates reaching 84%, while that of the left side remains at 33% [2]. Pseudomonas endocarditis is associated with high mortality. Our patient has invasive pseudomonas with several complications, including splenic infarct, renal infarct, and hemorrhagic stroke. Also, our patient had left-sided endocarditis without any other risk factor for left-sided endocarditis. 

Our patient had many characteristic findings of infective endocarditis. He had splinter hemorrhage and wedge-shaped infarcts in the spleen and kidneys, which are typical of infective endocarditis. Later on, during the course, he also developed a stroke, which was due to an embolic complication of infective endocarditis. 

For patients with Pseudomonas aeruginosa infective endocarditis, the regimen should include two anti-pseudomonal antibiotics from different classes to which the isolate is susceptible. Surgery is indicated for mobile vegetation greater than 10 mm, with or without embolic events [7]. Most patients with a cardioembolic stroke require long-term anticoagulation. Still, uncertainty exists regarding the best mode of starting long-term anticoagulation. However, anticoagulation of patients with cardioembolic stroke can be safely initiated shortly after an acute stroke.

## Conclusions

Pseudomonas aeruginosa is a rare cause of left-sided infective endocarditis in patients with a history of intravenous drug abuse. Our case presented with many findings that were characteristic of infective endocarditis, including splinter hemorrhage, splenic and renal infarct, and hemorrhagic embolus. Two anti-pseudomonal antibiotics are recommended for a total of at least six weeks. Surgery is recommended for vegetations greater than 10 mm.
